# Excessive Daytime Sleepiness and Its Associated Factors among Emergency Medicine Residents in South Korea: A Nationwide Survey

**DOI:** 10.1155/2021/6628361

**Published:** 2021-04-24

**Authors:** Song Yi Park, Kwang Hyun Cho, Ho Jung Kim, In Byung Kim, Bum Suk Seo, Suk Jae Choi, Yoo Sang Yoon, Donghune Key, Kyung Hye Park, Eu Sun Lee, Hyung Min Lee, Jiyoung Kim

**Affiliations:** ^1^Department of Emergency Medicine, College of Medicine, Dong-A University, Busan, Republic of Korea; ^2^Department of Emergency Medicine, Nowon Eulji Medical Center, Eulji University, Seoul, Republic of Korea; ^3^Department of Emergency Medicine, Soonchunhyang University Bucheon Hospital, Bucheon, Republic of Korea; ^4^Department of Emergency Medicine, Myongji Hospital, Goyang, Republic of Korea; ^5^Department of Emergency Medicine, Soonchunhyang University Hospital, Seoul, Republic of Korea; ^6^Department of Emergency Medicine, Elijah Hospital, Icheon, Republic of Korea; ^7^Department of Emergency Medicine, Inje University College of Medicine, Busan, Republic of Korea; ^8^Department of Emergency Medicine, Yeouido St. Mary's Hospital, Catholic University, Seoul, Republic of Korea; ^9^Department of Medical Education, Yonsei University Wonju College of Medicine, Wonju, Republic of Korea; ^10^Department of Emergency Medicine, Korea University Guro Hospital, Seoul, Republic of Korea; ^11^Department of Emergency Medicine, Kyunghee University Hospital, Seoul, Republic of Korea; ^12^Department of Neurology and Sleep Disorder Center, Bio Medical Research Institute, Pusan National University Hospital, Pusan National University School of Medicine, Busan, Republic of Korea

## Abstract

**Objective:**

Excessive daytime sleepiness (EDS) in emergency medicine (EM) residents is associated with patient safety. However, studies regarding EDS in EM residents are limited. The objective of this study was to identify the prevalence of EDS and its associated factors among EM residents.

**Methods:**

Epworth sleepiness scale scores, working hours per week (WHW), night working days per month, working environment, and depression were analyzed using data from the 2019 Korean Emergency Medicine Resident Survey.

**Results:**

The survey response rate was 63.8% (384/601). Among 241 respondents, the prevalence rate of EDS was 32.4%. Multivariable logistic regression analysis demonstrated that WHW (odds ratio [OR] = 1.03, 95% confidence interval [CI] = 1.01–1.06) and depression (OR = 3.64, 95% CI = 1.91–6.96) had increased ORs for EDS.

**Conclusions:**

Approximately one-third of EM residents had EDS. Depression and WHW were the associated factors.

## 1. Introduction

Emergency medical centers are places where lights are never switched off, whether they are crowded or not. Emergency physicians are always available for patients, whether or not the patients require them urgently. Working in night shifts may be a regular affair for emergency physicians. The same applies to emergency medicine (EM) residents.

Many studies have reported that night shift work is accompanied by various problems, including hypertension, coronary artery disease, substance abuse, and depression [1–5]. Sleep deprivation due to circadian rhythm changes caused by night shift work can cause insomnia and excessive daytime sleepiness (EDS). The effects of sleep loss are similar to those of alcohol ingestion [6]. According to Belayachi et al., nearly two-thirds of EM training physicians suffer from sleepiness [7]. A study by Steele et al. found that EM residents were involved in more motor vehicle collisions and near-crashes after a night shift than after other shifts while driving home [8].

EDS in EM residents at work could be more serious because it may potentially threaten patient safety. Although a study found that senior EM residents experienced no decrease in their ability to make accurate decisions in critical cases even after night shift work, a large number of residents perceived that sleep loss and fatigue had a major impact on their personal lives and abilities to perform their work. These findings raise concerns about the potential impact on residency training and patient safety [9, 10].

Legislation on restricted duty hours for residents has been implemented in many countries to encourage a superior-quality teaching environment and ensure safe patient care [11, 12]. In South Korea, an act for the improvement of training conditions and status of medical residents, enacted and enforced since 2016, ensures that residents are not forced to attend training programs for more than 80 hours per week, averaged over four weeks. In addition, the hours may be increased by 8 hours per week for educational purposes but for not more than 36 consecutive hours, and at least 10 duty off hours should be provided for rest after the consecutive training hours [13].

Studies have reported the use of psychostimulants and sedatives by EM residents to aid in night shift work and an association between self-apprehended sleepiness and quality of life [14, 15]. However, information about the prevalence of EDS and its associated factors in EM residents is currently limited. Therefore, the purposes of this study were to (1) investigate the prevalence of EDS and (2) identify the factors associated with EDS among EM residents.

## 2. Material and Methods

### 2.1. Study Setting and Subjects

We analyzed data from the Korean Emergency Medicine Resident Survey (KEMRS) conducted by the Korean Society of Emergency Medicine (KSEM) from August to October 2019. The committee of residency training of the KSEM evaluates the training programs and work environment of EM residents annually. However, there were concerns that their reports did not sufficiently reflect a picture of the training environment from the resident's perspective. Therefore, a task force team under the KSEM was organized to conduct a survey regarding the well-being and training environment of EM residents. The survey was distributed by postal mail and e-mail from August to September 2019.

**Figure 1:**
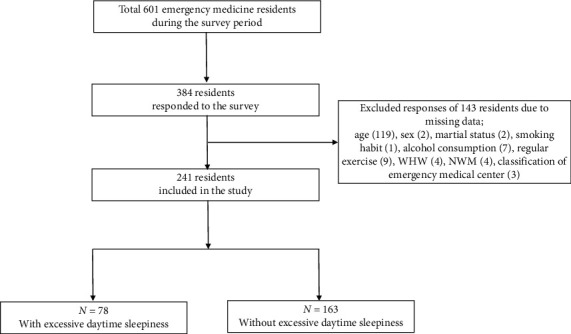
Flow chart of the study. WHW: working hours per week; NWM: night working days per month.

At the time the survey was conducted, there were 601 EM residents in South Korea to whom the questionnaires were distributed. 384 residents responded. However, there were missing data in age (30.99%), sex (0.52%), marital status (0.52%), smoking habit (0.26%), alcohol consumption (4.43%), regular exercise (2.60%), working hours per week (WHW) (1.04%), night working days per month (NWM) (1.04%), and level of emergency medical center they were training at (0.78%). Little's MCAR test for missing data resulted in a chi-square of 15.974 (df = 9, *p*=0.067) which indicates that these data were missing completely at random, and 143 cases were deleted by the complete case method. Finally, the responses of 241 EM residents were included in the analysis (Figure 1). This study was approved by the Institutional Review Board and Ethics Committee of Pusan National University Hospital (IRB No. H-2003-009-088).

### 2.2. Measures

Demographics (age, sex, marital status, smoking habit, alcohol consumption, and regular exercise) and variables in the following categories were assessed.

#### 2.2.1. Excessive Daytime Sleepiness

EDS was evaluated using the Epworth sleepiness scale (ESS), which is a widely used tool to assess daytime sleepiness. The ESS is a self-administered questionnaire with eight questions. Respondents were asked to rate their likelihood of falling asleep on a 4-point scale (0–3) while engaging in eight different activities. The ESS scores range from 0 to 24. The higher the ESS score, the higher the average sleep propensity in daily life or daytime sleepiness [16]. In the KEMRS, the Korean version of the ESS was used, which was previously validated in the Korean language [17]. An ESS score of 11 or more defined the presence of EDS [16, 18].

#### 2.2.2. Work Hours and Work Environment

To identify factors associated with EDS, WHW and NWM were assessed. For assessing WHW, respondents were asked “How many hours per week do you work on average?” WHW was collected as a continuous variable, ranging from 0 to 90 h. For assessing NWM, respondents were asked “How many nights do you work on an average every month?” The NWM was collected as a continuous variable, ranging from 0 to 20 days.

We presumed that the work environment would be related to EDS. Regional emergency medical centers have to take charge of more critical patients than local emergency medical centers do in South Korea. Thus, the level of emergency medical centers that the residents were training at was collected as an indicator of workload. Satisfaction with work schedule and availability of recesses were included as indicators of work quality. The recess is a short break, possible if there are alternative partners (not legally defined), and varies from hospital to hospital. The respondents were asked “How satisfied are you with your work schedule?” with the following options: (1) unsatisfied, (2) fair, and (3) satisfied and “do you have an official recess during night shifts?” with the following options: (1) no, (2) irregular, and (3) yes.

#### 2.2.3. Depression

Information on mental well-being was assessed using the Korean version of the Patient Health Questionnaire-9 (PHQ-9). The PHQ-9 is a self-reported test used to screen for depression and its severity. It consists of nine questions related to the criteria for depression. Each item was scored on a scale of 0–3 points, and the total score ranged from 0 to 27 points. Respondents who had scores of 10 or more on this measure were considered to have depression [19].

### 2.3. Statistical Analysis

Descriptive statistics were used to calculate the mean and standard deviation, and Student's t-test was used to compare continuous variables. A chi-square test was used to compare the categorical variables. A multivariable logistic regression analysis using the “Enter” method was conducted to calculate odds ratios (ORs) at 95% confidence intervals (CIs) to investigate the association between EDS and other variables. All statistical analyses were performed using SPSS version 25.0 (version 25.0; IBM, Armonk, NY, USA), and for all the tests, a *p* value <0.05 was considered statistically significant.

## 3. Results

### 3.1. Demographic Characteristics and Prevalence of EDS in EM Residents

**Figure 2:**
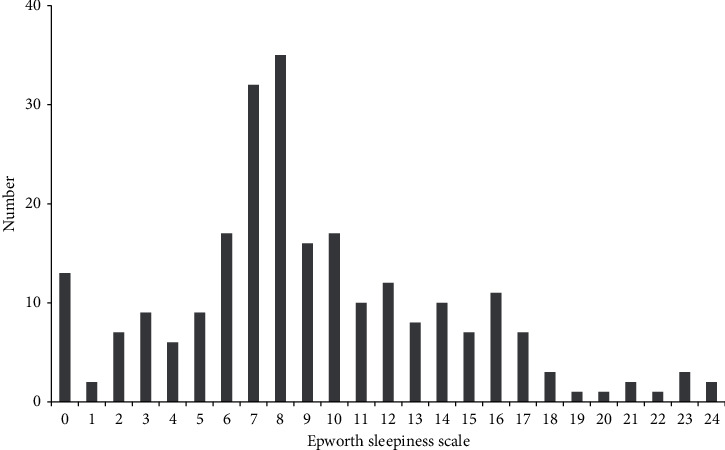
Distribution of the Epworth sleepiness scale scores among the emergency medicine residents.

Table 1 summarizes the basic demographic characteristics and work environment details. The mean WHW was 65.9 ± 12.0 h, and the mean NWM was 10.1 ± 3.5 days. The mean ESS score of respondents was 9.1 ± 5.1.  Figure 2 presents the distribution of the ESS scores, with “8” as the most frequent score (35/241, 14.5%), and 78 (32.4%) respondents were classified as having EDS.

### 3.2. Comparison of Demographic Characteristics and Work Environment in EM Residents according to EDS

The mean ESS score of EM residents with EDS was 15.0 ± 3.4 and those without EDS was 6.3 ± 2.8. The proportion of alcohol consumers and prevalence rate of depression was significantly higher in respondents with EDS than in those without EDS. Respondents with EDS had significantly higher WHW and NWM than those without EDS did. The proportion of those who were satisfied with their work schedule was significantly different between respondents with EDS and those without EDS. However, the proportion of availability of recesses was not significantly different between the two groups (Table 2).

### 3.3. Factors Associated with EDS

The multivariable analyses were conducted after adjusting for age, sex, marital status, smoking habit, alcohol consumption, regular exercise, level of emergency medical center, WHW, NWM, depression, satisfaction with work schedule, and availability of recess to investigate the factors associated with EDS. In the multivariable logistic analysis, WHW (OR = 1.03, 95% CI = 1.01–1.06) and depression (OR = 3.64, 95% CI = 1.91–6.96) were found to be associated with EDS (Table 3). NWM was found to have no association with EDS, as per the multivariable logistic regression model.

## 4. Discussion

The present study evaluated the prevalence of EDS among EM residents using the Korean version of the ESS. The main findings of our study were as follows: (1) the prevalence of EDS among EM residents was 32.4%, and (2) the psychological state of EM residents and average WHW were significantly associated with EDS.

EDS among the general Korean population and multiethnic Asian adults has been reported to be 12.2% and 9.2%, respectively [20, 21]. The prevalence of EDS among EM residents in our study was higher than that in the general population. According to previous studies, the prevalence of EDS among residents and attending physicians ranges widely. However, most studies have reported their EDS to be higher than that of the general population, and our results are consistent with their findings. According to a study on EDS among attending physicians by Singh et al., 15.9% of physicians were found to be sleepy with an ESS score greater than 10 [22]. A Japanese study involving residents of all medical specialties reported that 28.1% of residents suffered from excessive sleepiness [23]. Furthermore, in a study that investigated perceived sleepiness among Canadian anesthesia residents based on duty-call type, 50% of residents in the modified call group (12 or 16 h shift) and 45% residents in the traditional-call group (24h shift) showed an ESS score greater than 10 [24].

Many studies have reported an association between sleepiness and depression [25]. In a general population-based study, depression in young adults was associated with EDS [26]. Studies reporting sleep problems in night-shift workers have shown that depression is an associated factor [27]. The results are consistent with Carey's study, which found that sleep deprivation was related to depressive symptoms in firefighters [28]. In the present study, depression was associated with EDS. However, it was uncertain as to which problems, out of EDS, sleep deprivation, sleep maintenance, and poor sleep quality were more related to depression. Moreover, it was not established whether depression was the cause or the result of various sleep problems. Thus, we analyzed the mediation of depression on EDS and WHW. Baron and Kenny's three-stage mediation analysis was applied, and there was no mediating effect of depression or a possible mediating effect on EDS and WHW.

Few studies have reported an association between EDS and WHW and NWM in residents. The relationship between WHW, NWM, and EDS in EM residents has hardly been investigated. In a study on attending physicians, WHW was significantly correlated with EDS [22]. Wada et al. reported that there was no significant association between NWM and excessive sleepiness [23]. In general, it can be assumed that long WHW and frequent NWM are related to daytime sleepiness. However, in this study, it was found that WHW was related to EDS but NWM was not. This could be because the WHW of EM residents who participated in this study was limited to 80 hours or less, and a break time was guaranteed after night-shift work.

Although this is the first nationwide study on EDS and EM residents, it has several limitations because it is a secondary analysis of survey data. First, there is a possibility of nonrespondent bias, which is inherent to survey research. Second, 143 respondents were excluded from the study. Most of them did not respond to the question about their age. A sensitivity analysis was implemented to verify the pattern of the exclusion group, and we found differences in ESS scores, smoking, regular exercise, and satisfaction with work schedules between the two groups. Therefore, we performed a binary logistic regression analysis on the age nonresponse group to determine if these variables were related to ESS scores. However, no other variables were relevant except for depression (OR = 5.52, 95% CI = 1.87–16.24). This suggests that depression is highly related to ESS scores, even in the age nonresponse group. Third, only WHW and NWM were considered, and other details of the work environment, such as intensity of workload, were not considered. Only satisfaction with the work schedule was surveyed, and the details of the schedule were not analyzed. Studies have reported that consecutive night-shift workdays and shifting intervals appear to be related to sleep problems and poor health [29]. Fourth, this study investigated self-perceived EDS, and no objective assessment of sleep quality and sleep-related disorders among EM residents was conducted.

Despite these limitations, this study has several strengths. First, to the best of our knowledge, this is the first study to investigate the prevalence of EDS and identify associated factors among EM residents using a nationwide survey. Second, this study evaluated EDS in a homogenous group of EM residents who had to work night shifts due to the nature of their profession. Previous studies have been conducted on residents of various specialties. Third, this study included factors associated with EDS considering other aspects of the working environment, such as the level of training emergency medical centers and the availability of recess.

## 5. Conclusions

In this study, approximately one-third of the EM residents showed EDS, as evaluated by the ESS. EDS was associated with WHW and depression. EDS in EM residents could be directly related to patient safety. This demands constant close attention and surveillance for prevalence of EDS and a study of the factors associated with EDS.

## Figures and Tables

**Table 1:** Baseline demographic characteristics and work environment of the EM residents.

Variables	*N* = 241
*Age, years*	30.8 ± 3.3

*Sex*	
Male	169 (70.1%)
Female	72 (29.9%)

*Marital status*	
Unmarried	159 (66.0%)
Married	82 (34.0%)

*Smoking habit*	
Yes	46 (19.1%)
No	195 (80.9%)

*Alcohol consumption*	
Yes	146 (60.6%)
No	95 (39.4%)

*Regular exercise*	
Yes	86 (35.7%)
No	155 (64.3%)
*ESS*	9.1 ± 5.1
*WHW*	65.9 ± 12.0
*NWM*	10.1 ± 3.5

*Level of emergency medical center*	
Regional emergency center	120 (49.8%)
Local emergency center	121 (50.2%)

*Satisfaction of work schedule*	
Unsatisfied	69 (28.6%)
Fair	91 (37.8%)
Satisfied	81 (33.6%)

*Available of recess*	
No	147 (61.0%)
Irregular	38 (15.8%)
Yes	56 (23.2%)

*Depression*	
Yes	70 (29.0%)
No	171 (71.0%)

Variables are presented as mean ± standard deviation or number (%). EM, emergency medicine; ESS, Epworth sleepiness scale; WHW, working hours per week; NWM, night working days per month.

**Table 2:** Comparison of the demographic characteristics and work environment of the EM residents according to EDS.

Variables	Group with EDS (*N* = 78)	Group without EDS (*N* = 163)	*p*
*Age, years*	30.2 ± 3.1	31.1 ± 3.4	0.066

*Sex*			0.087
Male	49 (62.8%)	120 (73.6%)	
Female	29 (37.2%)	73 (26.4%)	

*Marital status*			0.304
Unmarried	55 (70.5%)	104 (63.8%)	
Married	23 (29.5%)	59 (36.2%)	

*Smoking habit*			0.087
Yes	10 (12.8%)	36 (22.1%)	
No	68 (87.2%)	127 (77.9%)	

*Alcohol consumption*			0.041
Yes	40 (51.3%)	106 (65.0%)	
No	38 (48.7%)	57 (35.0%)	

*Regular exercise*			0.094
Yes	22 (28.2%)	64 (39.3%)	
No	56 (71.8%)	99 (60.7%)	
*ESS*	15.0 ± 3.4	6.3 ± 2.3	<0.001
*WHW*	69.4 ± 12.1	64.2 ± 11.6	0.001
*NWM*	10.9 ± 3.8	9.8 ± 3.4	0.038

*Level of emergency medical center*			0.108
Regional emergency center	33 (42.3%)	87 (53.4%)	
Local emergency center	45 (57.7%)	76 (46.6%)	

*Satisfaction of work schedule*			0.033
Unsatisfied	30 (38.5%)	39 (23.9%)	
Fair	29 (37.2%)	62 (38.0%)	
Satisfied	19 (24.4%)	62 (38.0%)	

*Available of recess*			0.216
No	53 (67.9%)	94 (57.7%)	
Irregular	12 (15.4%)	26 (16.0%)	
Yes	13 (16.7%)	43 (26.4%)	

*Depression*			<0.001
Yes	38 (48.7%)	32 (19.6%)	
No	40 (51.3%)	131 (80.4%)	

Variables are presented as mean ± standard deviation or number (%). EM, emergency medicine; EDS, excessive daytime sleepiness; ESS, Epworth sleepiness scale; WHW, working hours per week; NWM, night working days per month.

**Table 3:** Analysis of associated factors of EDS in EM residents.

Variables	Beta	OR 95% CI (lower-upper)
*Age, years*	−0.07	0.93 (0.84–1.03)

*Sex*		
Male	0.65	1.00
Female		1.92 (0.98–3.76)

*Marital status*		
Unmarried	−0.18	1.00
Married		0.84 (0.41–1.70)

*Smoking habit*		
Yes	−0.53	0.59 (0.24–1.43)
No		1.00

*Alcohol consumption*		
Yes	−0.52	0.60 (0.32–1.13)
No		1.00

*Regular exercise*		
Yes	0.44	1.00
No		1.55 (0.79–3.06)
*WHW*	0.03	1.03 (1.01–1.06)
*NWM*	0.01	1.01 (0.92–1.12)

*Level of emergency medical center*		
Regional emergency center	0.60	1.00
Local emergency center		1.82 (0.94–3.52)

*Satisfaction of work schedule*		
Unsatisfied	0.53	1.70 (0.72–4.04)
Fair	−0.07	0.94 (0.42–2.07)
Satisfied		1.00

*Available of recess period*		
No	0.49	1.62 (0.68–3.90)
Irregular	0.55	1.73 (0.59–5.03)
Yes		1.00

*Depression*		
Yes	1.29	3.64 (1.91–6.96)
No		1.00

EM, emergency medicine; EDS, excessive daytime sleepiness; OR, odds ratio; CI, confidence interval. WHW: working hours per week, NWM: night working days per month.

## Data Availability

The data are available from the corresponding author on reasonable request.
